# Biomolecular alterations detected in multiple sclerosis skin fibroblasts using Fourier transform infrared spectroscopy

**DOI:** 10.3389/fncel.2023.1223912

**Published:** 2023-09-04

**Authors:** Jordan M. Wilkins, Oleksandr Gakh, Yong Guo, Bogdan Popescu, Nathan P. Staff, Claudia F. Lucchinetti

**Affiliations:** ^1^Department of Neurology, Mayo Clinic, Rochester, MN, United States; ^2^Department of Anatomy, Physiology, and Pharmacology, University of Saskatchewan, Saskatoon, SK, Canada; ^3^Cameco MS Neuroscience Research Center, University of Saskatchewan, Saskatoon, SK, Canada; ^4^Center for Multiple Sclerosis and Autoimmune Neurology, Mayo Clinic, Rochester, MN, United States

**Keywords:** amyotrophic lateral sclerosis, biomolecular profiling, Fourier transform infrared spectroscopy, multiple sclerosis, skin fibroblasts, sparse partial least squares-discriminant analysis

## Abstract

Multiple sclerosis (MS) is the leading cause of non-traumatic disability in young adults. New avenues are needed to help predict individuals at risk for developing MS and aid in diagnosis, prognosis, and outcome of therapeutic treatments. Previously, we showed that skin fibroblasts derived from patients with MS have altered signatures of cell stress and bioenergetics, which likely reflects changes in their protein, lipid, and biochemical profiles. Here, we used Fourier transform infrared (FTIR) spectroscopy to determine if the biochemical landscape of MS skin fibroblasts were altered when compared to age- and sex-matched controls (CTRL). More so, we sought to determine if FTIR spectroscopic signatures detected in MS skin fibroblasts are disease specific by comparing them to amyotrophic lateral sclerosis (ALS) skin fibroblasts. Spectral profiling of skin fibroblasts from MS individuals suggests significant alterations in lipid and protein organization and homeostasis, which may be affecting metabolic processes, cellular organization, and oxidation status. Sparse partial least squares-discriminant analysis of spectral profiles show that CTRL skin fibroblasts segregate well from diseased cells and that changes in MS and ALS may be unique. Differential changes in the spectral profile of CTRL, MS, and ALS cells support the development of FTIR spectroscopy to detect biomolecular modifications in patient-derived skin fibroblasts, which may eventually help establish novel peripheral biomarkers.

## 1. Introduction

Multiple sclerosis (MS) is a demyelinating disease of the central nervous system (CNS) and the leading cause of non-traumatic disability in young adults. Despite progress in treating relapsing MS, options for the progressive disease remains limited. Findings in human CNS tissue have greatly advanced our pathophysiological understanding of the disease (Lucchinetti et al., 2000; Reich et al., 2018; Tham et al., 2021). Several complex factors including genetics, environment, immune function, mitochondrial dysfunction, oxidative stress, lipid biosynthesis, and protein homeostasis are thought to contribute toward disease progression (Stone and Lin, 2015; Filippi et al., 2018; Pineda-Torra et al., 2021). However, the precise mechanisms are not fully understood. Thus, the development of additional approaches to study the pathophysiological basis of disease progression in MS are needed. More so, the identification of molecular biomarkers using peripherally accessible sources could help enhance personalized therapies in MS.

Skin fibroblasts from patients with neurological disorders including MS, Alzheimer’s disease (AD), Parkinson’s disease (PD), Huntington’s disease (HD), and amyotrophic lateral sclerosis (ALS) are proving to be useful for studying pathophysiological mechanisms and the development of biomarkers (Jędrak et al., 2018; Akerman et al., 2019; Romano et al., 2020; Wilkins et al., 2020). Previously, we identified altered stress and metabolic signatures in skin fibroblasts derived from individuals with MS (Wilkins et al., 2020). In MS skin fibroblasts, we detected apparent endoplasmic reticulum (ER) swelling when compared to control (CTRL) cells. When treated with hydrogen peroxide, MS skin fibroblasts had increased rates of death compared to CTRL cells. Additionally, the bioenergetics of MS skin fibroblasts were found to be perturbed compared to CTRL cells. Thus, we predict that the changes detected in MS skin fibroblasts are associated with biomolecular alterations likely affecting proteins and lipids. However, the extent of detectable biomolecular changes in MS skin fibroblasts remains to be specified. Therefore, we sought to determine if we could readily detect modified biomolecular processes in skin fibroblasts. To do so, we utilized Fourier transform infrared (FTIR) spectroscopy and chemometrics, which are effective methods to study biomolecular changes within biological materials (Mantsch and Jackson, 1995; Baker et al., 2014; Yang et al., 2022). The technique is reagent-free and non-destructive requiring minimal preparation prior to scanning. Molecules including lipids, proteins, nucleic acids (phosphate), and carbohydrates within biospecimens give unique vibrational frequencies, which change with structure, composition, and functional groups (Talari et al., 2017). Indeed, interrogation of skin fibroblasts and tissue by FTIR spectroscopy has detected altered biomolecules and processes that inform on aging, cancer, muscular dystrophy, quiescence, neurological disorders, and oxidation (Kosoglu et al., 2017; Kyriakidou et al., 2017; Eberhardt et al., 2018; Martel et al., 2020; Mateus et al., 2021; Martins et al., 2023). Therefore, characterization of biochemical changes of MS skin fibroblasts may inform on pathophysiological mechanisms involved in the disease, as well as provide a potential alternative site to detect and monitor early biomolecular alterations.

Neurological disorders including MS show features of mitochondrial dysfunction, protein aggregation, increased oxidative stress, and lipid degradation. Hence, research utilizing FTIR spectroscopy has demonstrated its application to detect macromolecular changes within diseased tissue. For instance, brain tissue derived from patients with AD displayed spectral signatures of lipid oxidation surrounding areas of amyloid plaques (Benseny-Cases et al., 2014). Spectral analysis of brain tissue from patients with PD suggest that Lewy bodies have an increased abundance of β-sheet structures on the periphery while lipids were more concentrated in the core (Araki et al., 2015). In MS brain tissue, altered spectral features were indicative of increased oxidation of proteins and lipids within white matter lesions (LeVine and Wetzel, 1998). Here, we tested whether skin fibroblasts derived from patients with MS have altered biomolecular profiles compared to CTRL cells as detected by FTIR spectroscopy. Previously, we showed that skin fibroblasts from patients with ALS had altered physiological properties when compared to control cells (Wilkins et al., 2020). Moreso, these changes were likely unique when compared to MS skin fibroblasts. Thus, we continued to utilize ALS skin fibroblasts in this study to evaluate disease specific biomolecular changes. We report that the use of FTIR spectroscopy coupled to multivariate analysis can detect changes in the biomolecular signatures of patient-derived skin fibroblasts and reliably segregate between MS, ALS, and CTRL individuals. Our results support the use of patient-derived skin fibroblasts for spectral phenotyping, which may aid in the understanding of pathophysiological mechanisms and the development of novel biomarkers enhancing individualized medicine approaches.

## 2. Materials and methods

### 2.1. Human skin fibroblasts

The usage of patient-derived skin fibroblasts in this study was approved by the Mayo Clinic Institutional Review Board. Skin fibroblasts obtained from CTRL individuals (no detectable CNS disorders) and patients diagnosed with ALS were obtained from the Mayo Clinic Center for Regenerative Medicine. Cells obtained from patients diagnosed with MS were obtained from the Mayo Clinic Center of Multiple Sclerosis and Autoimmune Neurology. Patient-derived skin fibroblasts were matched by age, sex, and passage number (Supplementary Table 1). All MS skin fibroblasts were collected from patients diagnosed with relapsing-remitting MS at the time of harvest. The time from diagnosis to skin fibroblast harvest ranged from 0.2 to 16.7 years in MS cells and 0.9 to 6.1 years in ALS cells (Supplementary Table 1). A total of 10 CTRL, 10 MS, and 10 ALS skin fibroblasts were used in this study (Supplementary Table 1). Any known family history of ALS is listed in Supplementary Table 1. Genotyping was not performed but does not exclude the possibility of possessing known genetic risk factors.

### 2.2. Reagents and materials

Minimum essential medium (MEM; cat. # 10-010-CV), phosphate-buffered saline (PBS; cat. # 21-031-CV), and MEM non-essential amino acids (NEAA; cat. # 25-025-CI) were purchased from Corning (New York, NY, USA). Trypsin (cat. # 25300), penicillin-streptomycin solution (PenStrep; cat. # 15140-122), and L-glutamine (L-Gln; cat. # 25030081) were purchased from Thermo Fisher Scientific (Waltham, MA, USA). Fetal bovine serum (FBS; cat. # F2442), silicone (cat. # Z273554), 10 mm × 10 mm cloning cylinders (cat. # CLS316610), and extracellular matrix gel (ECM gel; cat. # E1270) was purchased from Sigma-Aldrich (St. Louis, MO, USA). Low-e microscope slides were purchased from Kevley Technologies (cat. # CFR, Chesterland, OH, USA). Paraformaldehyde solution was purchased from Electron Microscopy Sciences (cat. # 15710, Hatfield, PA, USA).

### 2.3. Cell culture

Skin fibroblasts were incubated at 37°C in a humidified chamber with 5% CO_2_/95% air maintained in MEM media containing 10% FBS, 2 mM L-Gln, 1X NEAA, and 1X PenStrep. Cells were passaged when approximately 80% confluent. At plating for experiments, all cell lines were within five passages of each other (passages 7–11, Supplementary Table 1).

### 2.4. Sample preparation for spectroscopic analysis

Low-e slides were sterilized using 70% ethanol and air dried. Cloning cylinders were placed on the slide and sealed with silicone to prevent media from leaking. Each slide contained one CTRL, MS, and ALS skin fibroblast matched by age, sex, and passage number. Skin fibroblasts were detached from stock plates using trypsin, neutralized in media containing FBS, and spun. The supernatant was removed, and cells were suspended in media. A total of 20,000 cells in 200 μL of media were added per cloning cylinder. The slides were placed in a sterile 10 cm dish, covered, and incubated for 48 h. The media was removed, and skin fibroblasts were fixed in warm media containing 4% PFA for 10 min at 37°C. Cloning cylinders were removed and slides containing fixed cells were rinsed in PBS followed by Milli-Q water. Rinsed slides were dried in a glass desiccator containing desiccant for a minimum of 48 h.

### 2.5. Measurement by FTIR spectroscopy

All spectroscopic data was collected using an Agilent Cary 670 FTIR Spectrometer coupled to an Agilent Cary 620 FTIR Microscope (Agilent Technologies, Santa Clara, CA, USA) equipped with a liquid-N_2_ cooled detector. The microscope stage is enclosed with a custom-built chamber to displace the atmosphere with a nitrogen stream. A 7 × 7 tile mosaic was collected with 128 × 128 focal plane array detector. The spectral resolution was set to 8 cm^–1^ using a 25x objective with an optical resolution of 0.7 μm pixel size accumulating 80 scans per pixel. Measurements were done in reflection mode within the spectral region of 3500–950 cm^–1^. Background measurements (120 scans per pixel) were taken from a clean area (no cells) on the slide using the same acquisition parameters and subtracted from the respective spectra to compensate for atmospheric interference and instrument performance. Scanning was carried out using Agilent Resolutions Pro Software (Agilent Technologies, Santa Clara, CA, USA).

### 2.6. Spectral pre-processing

The initial preprocessing of raw spectral files was carried out using Quasar based on Orange software (Demsar et al., 2013; Toplak et al., 2021), which utilized Python 3.8 software. Supplementary Figure 1 represents the general workflow used for pre-processing. In summary, wavenumbers 2700–2000 cm^–1^ were removed due to the presence of a CO_2_ peak in the absorbance spectra and low signal contribution of biological material (Bruun et al., 2006; Song et al., 2019). All remaining spectra were baseline corrected using the rubber band method. Next, we used unsupervised clustering (*k*-Means) to separate spectra of cells from near background measurements. The resulting spectra was binned to reduce the file size for further processing. To help correct for potential Mie scattering (Bassan et al., 2010; Baker et al., 2014; Troein et al., 2020), we utilized the software Open Chemometrics Toolbox for Analysis and Visualization of Vibrational Spectroscopy (OCTAVVS) (Troein et al., 2020), which utilized Python 3.8 software. The OCTAVVS platform uses an algorithm for clustered resonant Mie scattering correction, which greatly reduces computational time while achieving similar performance to previously described models (Troein et al., 2020). The absorbance spectra are corrected against a reference spectrum, which is typically produced from a homogenous sample (e.g., casein, Matrigel, etc.) (Bassan et al., 2010; Troein et al., 2020). For our study, we generated a reference spectrum using ECM gel for use in the OCTAVVS software (Supplementary Figure 2). The corrected spectra were then smoothed (Savitzky-Golay, windows 5, polynomial order 2), averaged, and vector normalized in Orange software. The averaged absorbance spectra were then used to generate second derivative spectra (Savitzky-Golay, windows 7, polynomial order 2, derivative 2) and vector normalized in Orange software.

### 2.7. Data analysis

The second derivative curves were used to help identify wavenumber regions and peak positions that correspond to biochemical features previously described in the literature (Talari et al., 2017). As pre-processing can affect the presence of spectral bands, we compared the second derivative results reported herein with those using a Savitzky-Golay windows 15 and polynomial order 5. Two biologically relevant peaks (_*as*_N-CH_3_ and _*as*_CH_3_, listed in Table 1) were apparent using the current analysis, which were reduced in the latter (data not shown). However, as a Savitzky-Golay of windows 7 and polynomial order 2 has been used to analyze skin, breast, and tonsil material (Piqueras et al., 2015; Valle et al., 2021; Shakya et al., 2022), and a polynomial order 2 has been recommended for FTIR spectra (Morais et al., 2019), we deemed the current analysis to be suitable. The wavenumber regions identified (Table 1) were used to integrate the respective area under the absorbance spectra (Baker et al., 2014; Cakmak-Arslan et al., 2020; Ferreira et al., 2020; Szentirmai et al., 2020). Significant changes in the integrated areas between groups were detected using a one-way ANOVA with *post-hoc* Tukey’s test (GraphPad Prism 9). Tests for normality (Shapiro–Wilk) and homoscedasticity (Brown–Forsythe) indicate that the data largely have a normal distribution and equal variances, respectively. Apart from the amide A variable of MS skin fibroblasts, which had a Shapiro-Wilk test *P*-value = 0.0279, all other *P*-values were not significant (*P* > 0.05). Sparse partial least squares-discriminant analysis (sPLS-DA) was performed using the R package mixOmics (Rohart et al., 2017). All averaged second derivative data (10 CTRL, 10 MS, and 10 ALS) were used for generating the sPLS-DA classification model using centroid-based distances. Data was divided into two sets for sPLS-DA using M-fold cross-validation (3-fold) repeated with 200 iterations. The optimum number of components was predicted to be three with 60, 40, and 20 latent variables on the first three components, respectively. We further tested the sPLS-DA model with a 2- and 3-fold repeated cross validation utilizing the second derivative data generated with a Savitzky-Golay window of 15 and polynomial order 5. The results were consistent with the former analysis and did not affect the conclusions as presented herein (data not shown). The sPLS-DA plot, AUROC curve plot, correlation plot, and loading plots were generated using the mixOmics package.

**TABLE 1:** Assignments of absorbance spectra.

Region	Assignment	Wave-number
**Predominately protein**		
Amide A	*v*(N-H)	3315–3260 cm^–1^
Amide B	-NHCO-	3120–3000 cm^–1^
Amide I		1700–1580 cm^–1^
Amide II		1580–1480 cm^–1^
Amide II	δ_as_(N-CH_3_), choline	1500–1485 cm^–1^
Amide III		1300–1185 cm^–1^
**Predominately lipid**		
Total lipids		3000–2800 cm^–1^
Lipid asymmetric CH_3_	*v*_as_(CH_3_)	2972–2950 cm^–1^
Lipid asymmetric CH_2_	*v*_as_(CH_2_)	2936–2912 cm^–1^
Lipid symmetric CH_3_	*v*_s_(CH_3_)	2880–2865 cm^–1^
Lipid symmetric CH_2_	*v*_s_(CH_2_)	2860–2843 cm^–1^
Carbonyl ester	*v*(C = O)	1750–1725 cm^–1^
Acyl chain	δ(CH_2_), δ_as_(CH_3_)	1470–1430 cm^–1^
**Mixed region**		
Carbohydrates, lipids, proteins, phosphate (e.g., nucleic acids, phospholipids, phosphoproteins)	*v*_s_(C = O), C-O, *v*_as_(PO_2_^–^), *v*_s_(PO_2_^–^)	1300–1000 cm^–1^

## 3. Results

### 3.1. FTIR spectroscopy detects biological spectra in patient-derived skin fibroblasts

**FIGURE 1:**
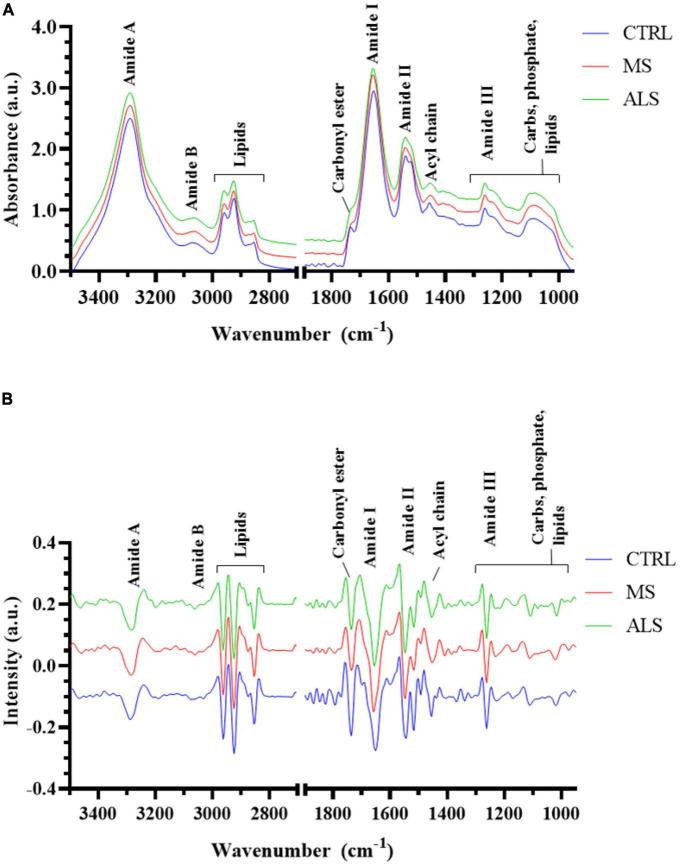
The spectral profiles of MS, ALS, and CTRL skin fibroblasts. **(A)** The average absorbance spectrum of MS, ALS, and CTRL skin fibroblasts (3500–950 cm^– 1^). **(B)** The average second derivative of the absorbance spectra of MS, ALS, and CTRL skin fibroblasts. For both graphs, MS and ALS spectra were offset for clarity. Regions corresponding to biological functional groups are highlighted.

The absorbance spectra for all skin fibroblasts (10 CTRL, 10 MS, and 10 ALS, Supplementary Table 1) were collected in the 3500–950 cm^–1^ range. All spectra were pre-processed using the same steps as outlined in the section “2. Materials and methods” and Supplementary Figure 1. The resulting absorption bands showed several characteristic peaks associated with biological samples (Figure 1A; Talari et al., 2017). To delineate specific wavenumber regions of interest for integrated analysis, we generated the second derivative spectra to help separate overlapping peaks (Figure 1B). Regions corresponding to amides, lipids, nucleic acids, and carbohydrates are highlighted in Figure 1 and listed in Table 1. Using the averaged second derivative spectra, multiple amide bands were detected including Amide A (3315–3260 cm^–1^), Amide B (3120–3000 cm^–1^), Amide I (1700–1580 cm^–1^), Amide II (1580–1480 cm^–1^), and Amide III (1300–1185 cm^–1^). Regions mainly associated with lipids were detected at 3000–2800 cm^–1^ (total lipids, symmetric and asymmetric CH_2_/CH_3_ vibrations), 1750–1725 cm^–1^ [carbonyl esters, *v*(C = O)], and 1470–1430 cm^–1^ [acyl chains, δ(CH_2_)] (Snyder et al., 1996; Oleszko et al., 2015). The wavenumbers from 1300 to 1000 cm^–1^ are composed of several metabolites and macromolecules including carbohydrates, proteins, lipids, and phosphate (e.g., nucleic acids, phospholipids, and phosphoproteins) (Colagar et al., 2011; Talari et al., 2017). These results demonstrate that patient-derived skin fibroblasts are suitable for generating spectral profiles as detected by FTIR spectroscopy.

### 3.2. Second derivative spectra are sufficient to segregate MS, ALS, and CTRL skin fibroblasts

**FIGURE 2:**
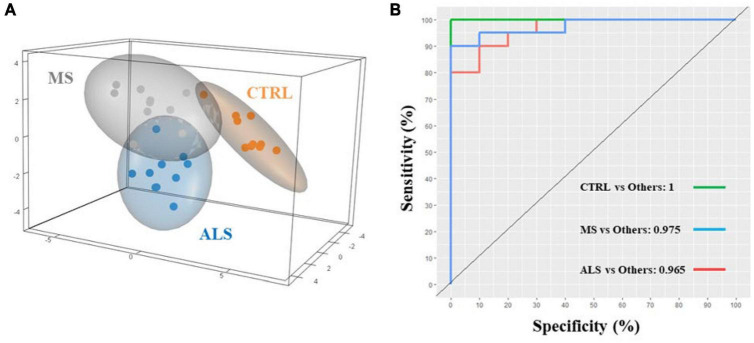
Multivariate analysis separates MS, ALS, and CTRL skin fibroblasts. The second derivative spectra of MS, ALS, and CTRL skin fibroblasts were used for sPLS-DA. **(A)** A 3D plot of the sPLS-DA analysis of MS, ALS, and CTRL skin fibroblasts. **(B)** An AUROC curve based on the performance of the cross-validated sPLS-DA model generated indicating good separation between groups.

In this study, we first aimed to determine if the spectral profiles from MS, ALS, and CTRL skin fibroblasts were unique as detected by FTIR spectroscopy. To do so, we analyzed the second derivative spectra (3500–2800 and 1800–950 cm^–1^) using sparse partial least squares-discriminant analysis (sPLS-DA) to determine if the skin fibroblasts could be effectively classified into their respective groups (Figure 2). The sPLS-DA model is particularly useful for classification of FTIR spectroscopy data where high multi-collinearity exists amongst the variables and the number of variables far outnumber the samples while focusing on the discrimination between groups (Chung and Keles, 2010; Lê Cao et al., 2011; Rohart et al., 2017). Optimization of the model suggests that three components with 60, 40, and 20 variables, respectively, were optimal for group separation (Supplementary Figure 3). A 3D plot of the three components shows reasonable separation between MS, ALS, and CTRL skin fibroblasts (Figure 2A). Performance of the model was evaluated using an area under the receiver operating characteristics (AUROC) curve. The AUROC curve values were 1, 0.975 and 0.965 for CTRL, MS, and ALS, respectively, indicating good separation between groups (Figure 2B). Thus, these results suggest that second derivative spectra from patient-derived skin fibroblasts may be useful for the prediction and classification of diseased cells from MS, ALS, and CTRL individuals.

### 3.3. Altered lipid, protein, and physiological profiles detected in MS skin fibroblasts

**FIGURE 3:**
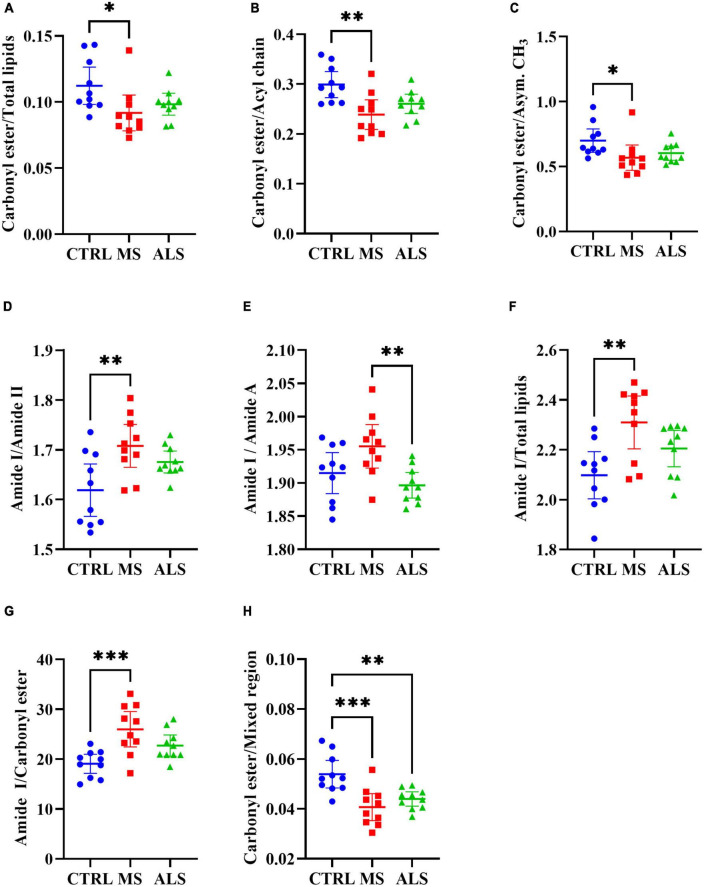
Physiological profiles are altered in MS and ALS skin fibroblasts. The integrated area under the absorbance spectra were used to identify informative quantitative ratios. The ratios include **(A)** carbonyl ester/total lipids, **(B)** carbonyl ester/acyl chain, **(C)** carbonyl ester/asymmetric CH_3_, **(D)** amide I/amide II, **(E)** amide I/amide A, **(F)** amide I/total lipids, **(G)** amide I/carbonyl ester, and **(H)** carbonyl ester/Mixed region. Significant changes between groups were detected using one-way ANOVA *post-hoc* Tukey’s test (*p < 0.05, ^**^p < 0.01, and ^***^p < 0.001). Integrated regions were as follows: Acyl chain, 1470–1430 cm^– 1^; Amide I, 1700–1580 cm^– 1^; Amide II, 1580–1480 cm^– 1^; Amide A, 3315–3260 cm^– 1^; Asymmetric CH_3_, 2972–2950 cm^– 1^; Carbonyl ester, 1750–1725 cm^– 1^; Mixed region, 1300–1000 cm^– 1^; and Total lipids, 3000–2800 cm^– 1^.

Quantitative ratios of the integrated absorbance spectra can provide insight into molecular changes occurring within tissue, cells, and biofluids, which have largely been described previously in the literature (Baker et al., 2014; Benseny-Cases et al., 2014; Talari et al., 2017). Additionally, ratios can help normalize and minimize the effect of experimental artifacts. Using the band regions identified from the second derivative spectra (Table 1), we calculated ratios of the respective integrated area under the absorbance spectrum (Figure 3 and Supplementary Table 2). We identified several lipid ratios that were significantly altered in MS and/or ALS skin fibroblasts compared to CTRL cells. The ratios of carbonyl ester/total lipids (∼1740/3000–2800 cm^–1^), carbonyl ester/acyl chain (∼1740/1450 cm^–1^), and carbonyl ester/asymmetric CH_3_ (∼1740/2960 cm^–1^) were significantly decreased in MS skin fibroblasts when compared to CTRL cells (Figures 3A–C). These altered ratios suggest that lipid homeostasis is perturbed, which may include abundance, membrane organization, structure, and peroxidation (Benseny-Cases et al., 2014; Oleszko et al., 2015; Barraza-Garza et al., 2016; Dreier et al., 2019). Interestingly, analysis of the integrated area under the absorbance spectra for total lipids, carbonyl ester, and acyl chains suggest an overall decrease in cellular lipids for MS and ALS skin fibroblasts when compared to CTRL cells (Supplementary Figures 4A–C). Additionally, ratios previously described to inform on membrane polarity (∼2920/2870 cm^–1^), lipid chain packing (∼2850/2870 cm^–1^), the degree of lipid saturation (∼2920/2960 cm^–1^), and phospholipid chain length (∼2920/3000–2800 cm^–1^) were further evaluated, however, we did not detect any significant changes (Supplementary Figures 4D–G; Saeed et al., 2015). These observations suggest that lipid abundance is reduced likely affecting cellular membrane organization, metabolism, signaling, and oxidative stress via peroxidation.

Similar to lipids, protein dysregulation in MS is well described (Andhavarapu et al., 2019). We analyzed several quantitative ratios related to protein dyshomeostasis. The ratio of Amide I to Amide II (1700–1580/1580–1480 cm^–1^) revealed a significant increase in MS skin fibroblasts compared to CTRL cells (Figure 3D). A change in the Amide I/Amide II ratio is largely attributed to structural changes in proteins (Ricciardi et al., 2020). A general rise in protein abundance was detected in MS skin fibroblasts compared to ALS and CTRL cells as detected by an increase in the Amide I region (Supplementary Figure 4H) while Amide II levels were down in ALS (Supplementary Figure 4I). Interestingly, the ratio between Amide I and Amide A revealed a significant increase in MS compared to ALS (Figure 3E). Amide A largely arises from N-H stretching of peptide linkages, which is thought to arise from the overtone of the Amide II, and/or interactions between Amide I and Amide II, reflecting possible disorder in protein secondary structures (Krimm and Dwivedi, 1982; Magazù et al., 2012). Thus, the Amide I/Amide A ratio may be reflecting distinct protein structural changes in MS when compared to ALS skin fibroblasts. Overall, these findings suggest that protein structure, organization, and abundance are perturbed in MS and ALS skin fibroblasts compared to CTRL cells.

Lastly, we explored ratios linked to the status of membrane organization, protein folding, and cellular physiology (Szalontai et al., 2000; Colagar et al., 2011; Cakmak et al., 2012; El Khoury et al., 2019; Poonprasartporn and Chan, 2021). The Amide I/Total Lipids ratio resulted in a significant increase in MS skin fibroblasts compared to CTRL cells (Figure 3F). This reflects an imbalance in protein and lipid homeostasis. Similarly, the Amide I/carbonyl ester is significantly increased in MS skin fibroblasts (Figure 3G), which may affect membrane organization and protein folding and dynamics. The region 1300–1000 cm^–1^ is comprised of lipids, proteins, phosphate, and carbohydrates reflecting several metabolites and macromolecules (Colagar et al., 2011; Caine et al., 2012). The ratio of carbonyl ester/1300−1000 cm^–1^ was found to be significantly decreased in both MS and ALS skin fibroblasts compared to CTRL cells suggesting diseased cells are biochemically distinct (Figure 3H). Taken together, these observations indicate that the physiological status of diseased skin fibroblasts are unique suggesting that cellular membranes, protein dynamics, oxidative stress, and metabolite profiles are perturbed.

### 3.4. Second derivative spectra reveal altered protein and lipid structure in MS and ALS skin fibroblasts

**FIGURE 4:**
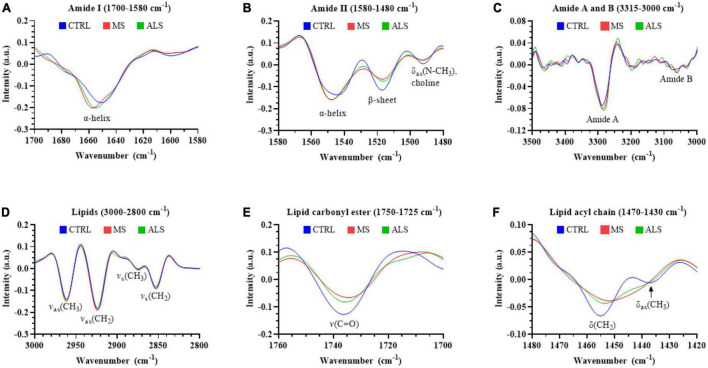
The second derivative of the absorbance spectra reveals changes in the biomolecular profiles of MS and ALS skin fibroblasts compared to CTRL cells. The average second derivative of the absorbance spectra of MS, ALS, and CTRL skin fibroblasts is shown. The second derivative spectra of various regions corresponding to biological functional groups including **(A)** amide I (1700–1580 cm^– 1^), **(B)** amide II (1580–1480 cm^– 1^), **(C)** amide A and B (3315–3260 cm^– 1^ and 3120–3000 cm^– 1^, respectively), **(D)** total lipids (3000–2800 cm^– 1^), **(E)** carbonyl ester (1750–1725 cm^– 1^), and **(F)** acyl chain (1470–1430 cm^– 1^). Standard deviations of the spectra can be seen in Supplementary Figure 5.

The second derivative spectra are particularly useful to interrogate shifts in the peak location and shape, which can help inform on biomolecular alterations within the biospecimens. Therefore, we compared the mean second derivative spectra of MS, ALS, and CTRL skin fibroblasts (Figure 4). Notably, we detected a shift in the peak positions of the Amide I and Amide II bands (Figures 4A, B, respectively). The Amide I band near the α-helical region (∼1650 cm^–1^) in MS and ALS shifted toward higher wavenumbers when compared to CTRL skin fibroblasts (Figure 4A). This suggests an increase in β-turn structures in both MS and ALS cells, which may be indicative of protein alterations commonly associated with neurological disorders including MS and ALS (David and Tayebi, 2014; McAlary et al., 2019; Ricciardi et al., 2020). In the Amide II region, we detected three peaks (Figure 4B). The peaks near 1545 and 1520 cm^–1^ are largely associated with α-helixes and β-sheets, respectively (Murphy et al., 2014). The third peak around 1490 cm^–1^ is less clear but may be related to additional β-structures or choline groups of lipids (Casal and Mantsch, 1984; Murphy et al., 2014; Oleszko et al., 2015). In agreement with the shift in the α-helical region of the Amide I peak, the α-helix peak detected in the Amide II region was also shifted toward higher wavenumbers in MS and ALS when compared to CTRL skin fibroblasts further suggesting altered secondary structures in the diseased cells (Figure 4B). The Amide A and Amide B peaks were found between ∼3350 and 3000 cm^–1^ (Figure 4C). While the Amide B peak was relatively weak, we observed a prominent band for Amide A. An apparent increase in the Amide A intensity and shift toward lower wavenumbers was observed in both MS and ALS skin fibroblasts when compared to CTRL cells (Figure 4C), which is likely associated with the α-helix shifts observed in the Amide I and II regions (Magazù et al., 2012). Four peaks were observed in the lipid region (3000–2800 cm^–1^) mainly arising from C-H stretching including the symmetric CH_2_ [*v*_*s*_(CH_2_), 2860–2843 cm^–1^], symmetric CH_3_ [*v*_*s*_(CH_3_), 2880–2865 cm^–1^], asymmetric CH_2_ [*v*_*as*_(CH_2_), 2936–2912 cm^–1^], and asymmetric CH_3_ [*v*_*as*_(CH_3_), 2972–2950 cm^–1^] peaks (Figure 4D). Two additional peaks, carbonyl ester and acyl chain, were detected in skin fibroblasts, which are thought to arise predominately from lipids (Derenne et al., 2013). The carbonyl ester [*v*(C = O)] was detected around 1735 cm^–1^ and the acyl chain near 1450 cm^–1^ (Figures 4E, F, respectively). The acyl chain of CTRL cells reveals two peaks likely representing the bending/scissoring of CH_2_ [∼1455 cm^–1^, δ(CH_2_)] and deformation mode of methyl groups [∼1435 cm^–1^, δ_as_(CH_3_)] (Casal and Mantsch, 1984; Ami et al., 2014). Interestingly, MS and ALS skin fibroblasts have a single acyl chain peak possibly indicating altered organization, structure, and packing of the acyl chains in lipid membranes (Figure 4F; Casal and Mantsch, 1984; Ami et al., 2014). Overall, the second derivative spectra suggests that protein structure and lipid organization are altered in MS and ALS cells when compared to CTRL skin fibroblasts.

## 4. Discussion

Using patient-derived skin fibroblasts, we determined that the physiological properties in MS and ALS cells have unique spectroscopic profiles that may be suitable for the development of novel peripheral biomarkers. We previously demonstrated that stress signatures and bioenergetics in MS and ALS cells were perturbed when compared to CTRL individuals (Wilkins et al., 2020). Here, evaluation using FTIR spectroscopy coupled to multivariate analysis strongly suggests the spectral profiles of MS, ALS, and CTRL individuals are unique. More so, examination of their second derivative spectra and use of quantitative ratios suggest that distinct alterations in MS and ALS skin fibroblasts relating to lipid and protein homeostasis, structure, and function are disrupted when compared to CTRL cells. These findings support the use of MS skin fibroblasts to study pathophysiological mechanisms and for the development of novel peripheral biomarkers.

Previously, in MS skin fibroblasts, we detected an apparent increase in ER swelling compared to CTRL cells, which may be indicative of increased cell stress affecting lipid and protein homeostasis among other physiological processes (Stone and Lin, 2015; Andhavarapu et al., 2019; Wilkins et al., 2020). When treated with hydrogen peroxide, MS skin fibroblasts had reduced cell survival rates compared to both ALS and CTRL cells suggesting that processes controlling oxidative stress in MS skin fibroblasts were altered. Furthermore, mitochondrial and glycolytic metabolic functions in MS skin fibroblasts were perturbed compared to CTRL cells, which is often associated with increased stress and altered biological processes (Hotamisligil and Davis, 2016). Thus, based on our previous findings, it is conceivable that biomolecular modifications and altered lipid and protein homeostasis exist in MS skin fibroblasts. This is in line with our current observations that spectral profiles in MS, ALS, and CTRL skin fibroblasts are unique and indicative of altered physiological profiles in diseased cells.

Studies in other labs have also demonstrated that diseased tissues have altered biomolecular profiles as detected by FTIR spectroscopy. Brain tissue from MS and CTRL individuals suggested that white matter lesions had an increase in the carbonyl ester to acyl chain (1740 to 1468 cm^–1^) ratio and broadening of the Amide I peak. These changes in MS lesions were thought to be due to lipid and protein peroxidation, respectively (LeVine and Wetzel, 1998). In our study, we observed a significant decrease in the carbonyl ester to acyl chain ratio in MS skin fibroblasts compared to CTRL cells. Likewise, we detected shifting of the α-helical region of the Amide I and Amide II peak suggestive of altered protein structures in MS and ALS skin fibroblasts when compared to CTRL cells. Additional investigations using FTIR spectroscopy correlated an increase in the ratio of 1740/2960 cm^–1^ as a measurement of lipid oxidation and a sign of oxidative stress (Benseny-Cases et al., 2014; Oleszko et al., 2015; Barraza-Garza et al., 2016). Interestingly, in our current study, we found the ratio of 1740/2960 cm^–1^ to be significantly lower in MS cells compared to CTRL skin fibroblasts, which is in agreement with the decrease in the carbonyl ester/acyl chain ratio.

Oxidative stress has long been implicated in MS due to the inflammatory nature of the disease and other factors including mitochondrial dysfunction (van Horssen et al., 2008; Mahad et al., 2015). Several antioxidant mechanisms exist to control the level oxidation within a cell. However, an increase in oxidative stress can result in the oxidation of proteins, lipids, and nucleic acids resulting in altered structure and function. Additionally, increased lipid peroxidation can result in lipid acyl chain disorder and the degradation of lipids and cellular membranes (Lamba et al., 1991). Indeed, an overall decrease of lipid abundance as detected by FTIR spectroscopy in MS skin fibroblasts was observed. More so, the lipid acyl chain peak (∼1460 cm^–1^) had two peaks in CTRL skin fibroblasts while MS and ALS cells presented with a single peak. This may be reflecting disorder caused by modification of lipid acyl chains in MS and ALS. Overall, these observations may indicate changes in oxidative stress homeostasis of MS skin fibroblasts resulting in altered lipid organization and abundance. Furthermore, protein misfolding and dyshomeostasis are characteristic features of MS (Stone and Lin, 2015; Andhavarapu et al., 2019). Studies using FTIR spectroscopy have further described altered protein homeostasis in various biospecimens from MS patients including blood plasma and serum (Kołodziej et al., 2022), cerebrospinal fluid (Yonar et al., 2018), and brain tissue (LeVine and Wetzel, 1998). In each study, the general conclusion is that protein modifications, structure, and aggregation are perturbed in MS biospecimens vs. CTRL samples. This likely reflects a common endpoint inherent to MS (e.g., oxidative stress, mitochondrial dysfunction, etc.). In MS skin fibroblasts, significant changes in various amide peaks were observed when compared to CTRL and ALS skin fibroblasts. This likely reflects an overall disruption in protein secondary structures and abundance. Similarly, several shifts in the amide peaks (I, II, and A) of MS and ALS skin fibroblasts were observed supporting the notion that diseased skin fibroblasts have altered protein structures. In a similar study using serum from MS, ALS, and CTRL individuals, the region 1200–1000 cm^–1^ was identified as the most differential amongst the groups (El Khoury et al., 2019). This was predicted to be due to changes in carbohydrates and nucleic acids of MS and ALS serum samples. Interestingly, in this study, the ratio of carbonyl ester to the region of 1300–1000 cm^–1^ was significantly altered in MS and ALS when compared to CTRL individuals. As the region of 1300–1000 cm^–1^ contains several metabolites and macromolecules including lipids, proteins, nucleic acids (phosphate), and carbohydrates, this ratio likely reflects a unique metabolic and physiological status in MS and ALS skin fibroblasts.

Ultimately, our results support the use of FTIR spectroscopy to detect biomolecular alterations in MS skin fibroblasts, which may be suitable for the development of novel biomarkers and studying pathophysiological mechanisms. While the change of specific ratios in skin fibroblasts did not match precisely with previously reported results, it is likely due to differences in the biospecimens used (e.g., blood, brain tissue, cerebrospinal fluid) and experimental setup. Additionally, multivariate analysis strongly supports the feasibility of using second derivative spectra for the classification of patient-derived skin fibroblasts. However, larger cohorts will need to be tested to also address potential confounding factors (e.g., age, sex, medications, disease status, genetic variants, etc.), and to confirm the segregation of data as presented here using a separate training and testing group. Nonetheless, our results suggest that MS is a CNS disorder that which contributes to biochemical alterations detected in the periphery that can be interrogated using FTIR spectroscopy of patient-derived skin fibroblasts.

## 5. Conclusion

Taken together, our findings demonstrate that skin fibroblasts derived from patients with MS have inherent disease-associated changes resulting in unique biomolecular profiles as detected by FTIR spectroscopy. Establishing distinct profiles in MS skin fibroblasts have the potential to advance the development of novel biomarkers facilitating spectral phenotyping, which may inform pathophysiological mechanisms as well as aid in disease monitoring. The results of this study suggest that lipid and protein homeostasis are perturbed in MS skin fibroblasts with changes indicative of altered physiological properties involved in modulating oxidative stress, metabolic function, and cellular organization. Thus, interrogating patient-derived skin fibroblasts may provide a unique opportunity to study pathophysiological mechanisms that could become targets for future therapies or a platform for establishing a skin-brain axis in MS.

## Data availability statement

The raw data supporting the conclusions of this article will be made available by the authors, without undue reservation.

## Ethics statement

The studies involving humans were approved by the Mayo Clinic Institutional Review Board. The studies were conducted in accordance with the local legislation and institutional requirements. The participants provided their written informed consent to participate in this study.

## Author contributions

JW, OG, and CL conceptualized the study. JW and OG performed experiments and acquired the data. NS contributed new reagents. JW performed data analysis and drafted the manuscript. All authors contributed to the interpretation of results and revising of the article for intellectual content.
