# Supplementary material for: Biomolecular alterations detected in multiple sclerosis skin fibroblasts using Fourier transform infrared spectroscopy
Source: Front Cell Neurosci. 2023 Sep 4;17:1223912. doi: 10.3389/fncel.2023.1223912 (PMC10512183; doi:10.3389/fncel.2023.1223912)
Supplement: Supplementary file 1 [file Data_Sheet_1.docx]

Supplementary Material

Biomolecular alterations detected in multiple sclerosis skin fibroblasts using Fourier transform infrared spectroscopy

Jordan M. Wilkins, Oleksandr Gakh, Yong Guo, Bogdan Popescu, Nathan P. Staff, Claudia F. Lucchinetti

*** Correspondence:**Claudia Lucchinetti:clucchinetti@mayo.edu

**Supplementary Figure 1**


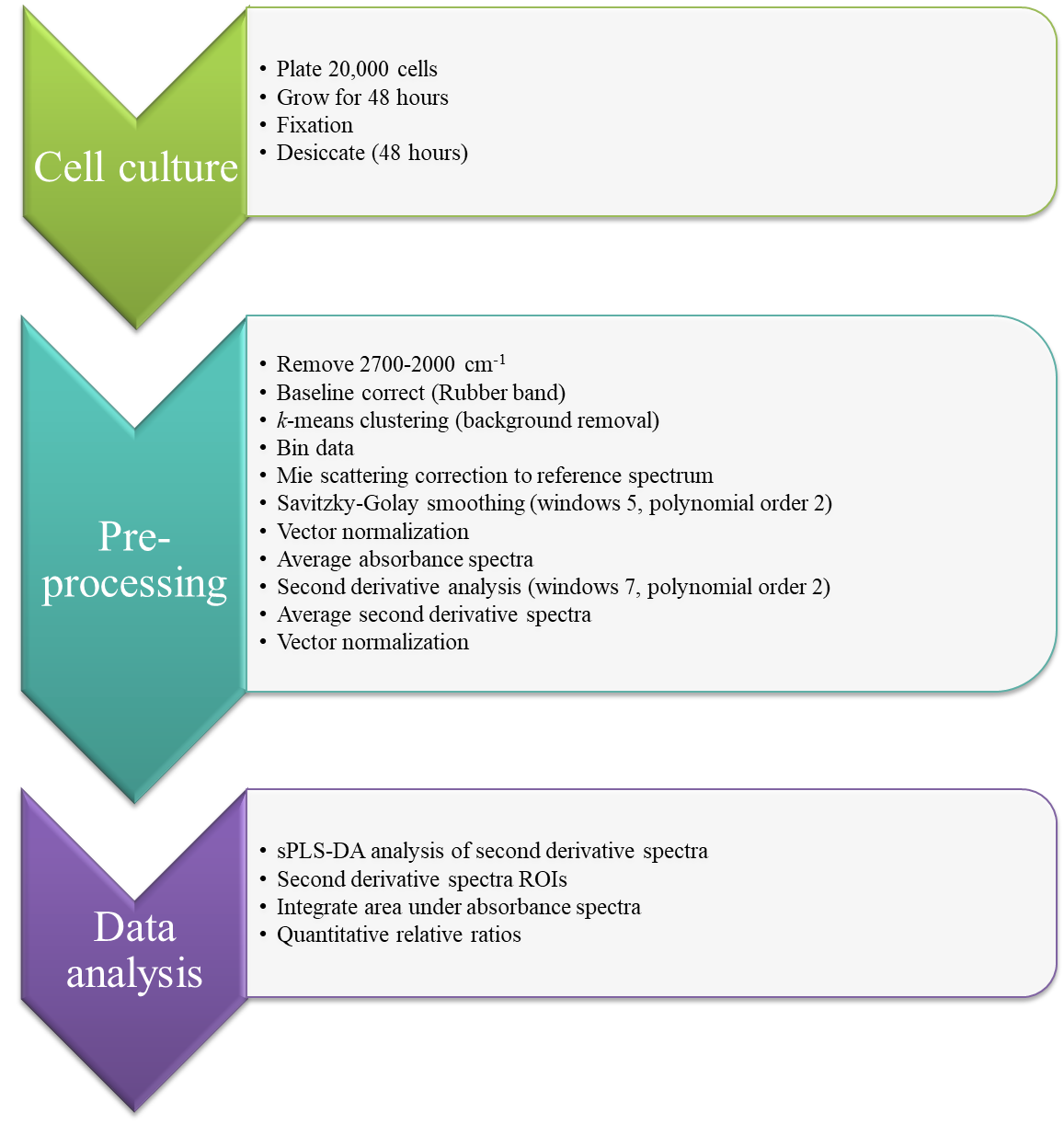


**Supplementary Figure 1. Workflow used in study.** The overall steps performed in this study including cell culture, pre-processing of spectral data, and data analysis.

**Supplementary Figure 2**

**
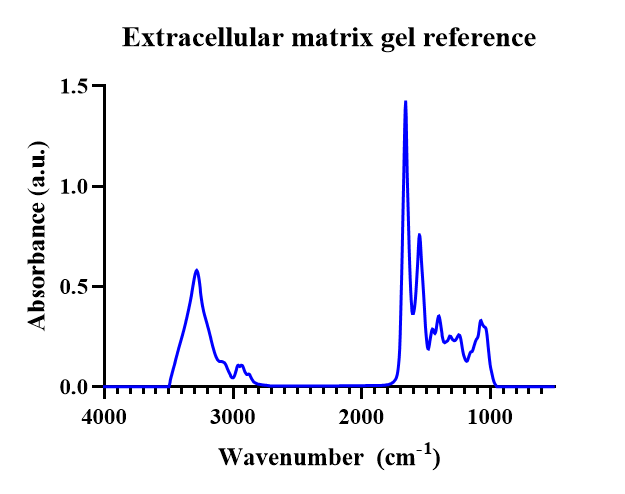
**

**Supplementary Figure 2. Reference spectra for correcting Mie scattering.** Extracellular matrix (ECM) gel was spotted on Low-e slides and scanned by FTIR spectroscopy using the same setup described in materials and methods. The ECM absorbance spectra were averaged and used as a reference for Mie scattering correction in the Open Chemometrics Toolbox for Analysis and Visualization of Vibrational Spectroscopy (OCTAVVS) software.

**Supplementary Figure 3**


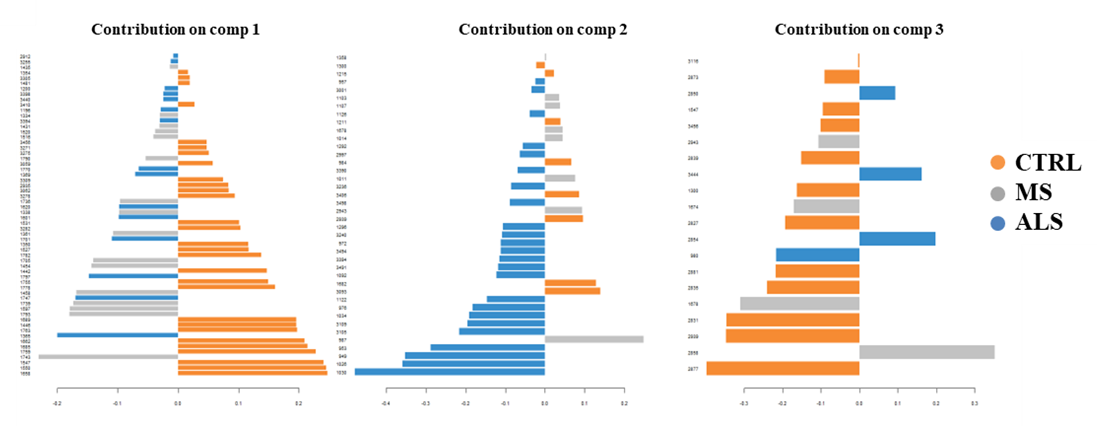


**Supplementary Figure 3. Variable loadings used for classifying patient-derived skin fibroblasts.** The second derivative spectra of MS, ALS, and CTRL skin fibroblasts were used for sPLS-DA.Tuning of the sPLS-DA model using repeated cross-validation predicted three components with 60, 40, and 20 variables, respectively.Loading plots showing the contribution of each variable on the respective components is shown.

**Supplementary Figure 4**


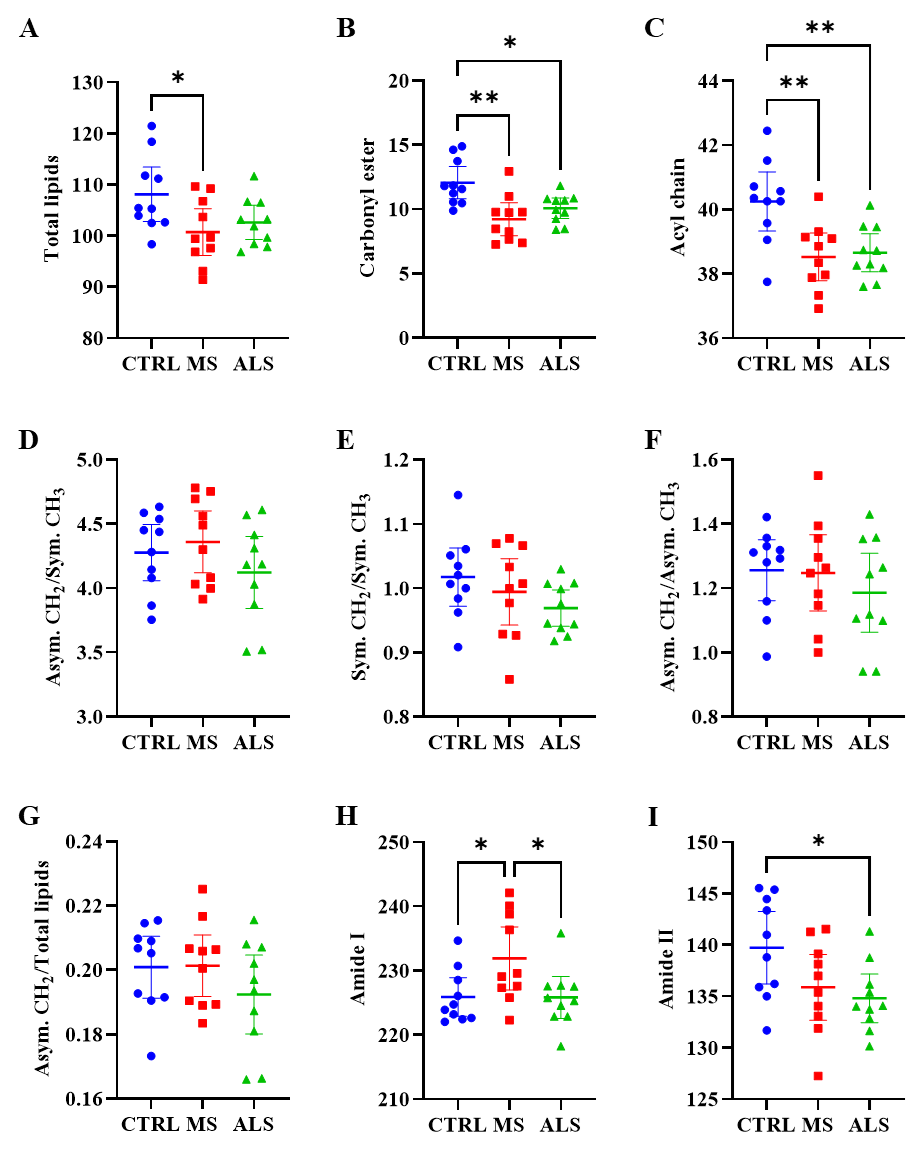


**Supplementary Figure 4. Integrative and ratiometric analysis of MS, ALS, and CTRL skin fibroblasts.** Area under the absorbance spectra was integrated for various regions of interest and used for quantitative ratio analysis. (A, B, C, H, and I) Integrated areas under the absorbance spectra suggestive of altered abundances in lipids and proteins of MS and/or ALS skin fibroblasts compared to CTRL cells. (D, E, F, and G) Quantitative ratios previously described to inform on lipid properties including (D) membrane polarity, (E) lipid chain packing, (F) degree of saturation, and (G) phospholipid chain length. Significant changes between groups were detected using one-way ANOVA post hoc Tukey test (* p < 0.05 and ** p < 0.01). Integrated regions were as follows: Acyl chain, 1470-1430 cm^-1^; Amide I, 1700-1580 cm^-1^; Amide II, 1580-1480 cm^-1^; Asymmetric CH_2_, 2936-2912 cm^-1^; Asymmetric CH_3_, 2972-2950 cm^-1^; Carbonyl ester, 1750-1725 cm^-1^; Symmetric CH_2_, 2860-2843 cm^-1^; Symmetric CH_3_, 2880-2865 cm^-1^; and Total lipids, 3000-2800 cm^-1^.

**Supplementary Figure 5**


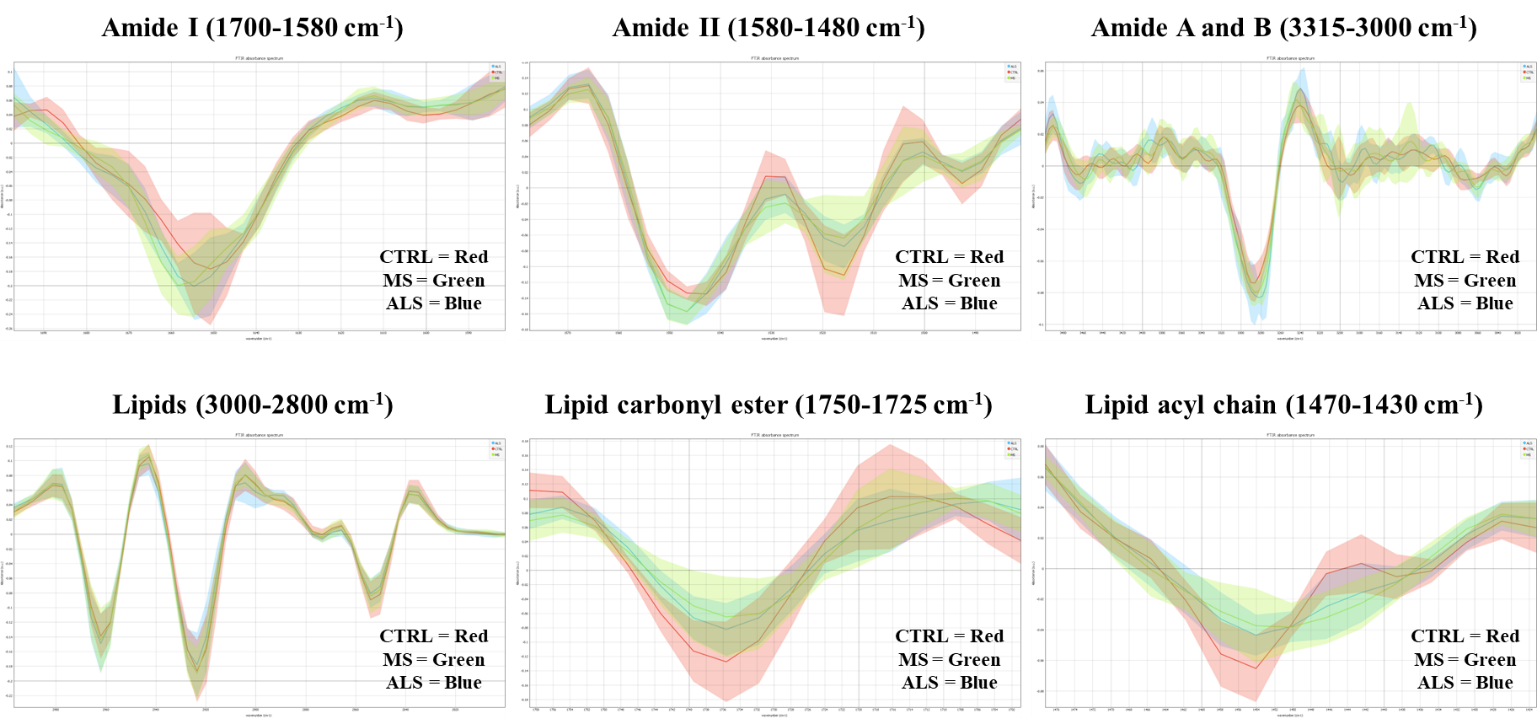


**Supplementary Figure 5. Second derivative spectra with standard deviations.** The average second derivative spectra as shown in Figure 4. Here, the standard deviations are shown as shaded regions on the graph.

**Supplementary Table 1 – Skin fibroblasts used in study**

| Biotrust ID | Age | Sex | Diagnosis^a^ | Years from diagnosis^b^ | Passage^c^ | Family history^d^ |
| --- | --- | --- | --- | --- | --- | --- |
| 014-BIOTR-0022 | 33 | Female | Normal | NA | 8 | NA |
| 014-BIOTR-0028 | 39 | Female | Normal | NA | 8 | NA |
| 100-BIOTR-0012 | 54 | Female | Normal | NA | 8 | NA |
| 100-BIOTR-0026 | 65 | Female | Normal | NA | 8 | NA |
| 100-BIOTR-0034 | 70 | Female | Normal | NA | 9 | NA |
| 014-BIOTR-0015 | 38 | Male | Normal | NA | 10 | NA |
| 100-BIOTR-0020 | 44 | Male | Normal | NA | 9 | NA |
| 019-BIOTR-0225 | 51 | Male | Normal | NA | 8 | NA |
| 100-BIOTR-0027 | 59 | Male | Normal | NA | 8 | NA |
| 100-BIOTR-0033 | 68 | Male | Normal | NA | 8 | NA |
| **Average (yr)** | **52.1** | **± 12.6** |  |  |  |  |
| 019-BIOTR-0009 | 32 | Female | MS | 3.1 | 8 | NA |
| 019-BIOTR-0014 | 39 | Female | MS | 3.4 | 11 | NA |
| 019-BIOTR-0019 | 51 | Female | MS | 10.1 | 10 | NA |
| 019-BIOTR-0021 | 66 | Female | MS | 1.9 | 9 | NA |
| 019-BIOTR-0030 | 70 | Female | MS | 14.9 | 9 | NA |
| 019-BIOTR-0040 | 36 | Male | MS | 1.5 | 8 | NA |
| 019-BIOTR-0028 | 44 | Male | MS | 0.2 | 10 | NA |
| 019-BIOTR-0068 | 50 | Male | MS | 14.3 | 8 | NA |
| 019-BIOTR-0029 | 59 | Male | MS | 16.7 | 8 | NA |
| 019-BIOTR-0055 | 72 | Male | MS | 11.5 | 8 | NA |
| **Average (yr)** | **51.9** | **± 13.6** |  |  |  |  |
| 009-BIOTR-0020 | 33 | Female | ALS | 2.7 | 8 |  |
| 009-BIOTR-0018 | 42 | Female | ALS | 0.9 | 7 | Yes |
| 009-BIOTR-0017 | 56 | Female | ALS | 6.1 | 7 |  |
| 009-BIOTR-0013 | 62 | Female | ALS | 1.6 | 8 |  |
| 009-BIOTR-0059 | 72 | Female | ALS | 4 | 8 |  |
| 009-BIOTR-0060 | 36 | Male | ALS | 2.5 | 8 |  |
| 009-BIOTR-0033 | 45 | Male | ALS | 1.8 | 8 |  |
| 009-BIOTR-0029 | 52 | Male | ALS | 2 | 8 |  |
| 009-BIOTR-0032 | 63 | Male | ALS | 0.9 | 8 | Yes |
| 009-BIOTR-0038 | 71 | Male | ALS | 3 | 8 | Yes |
| **Average (yr)** | **53.2** | **± 13.2** |  |  |  |  |
| ^a^ Diagnosis is at time of skin fibroblast collection | | | | |  |  |
| ^b^ Years from diagnosis to skin fibroblast harvest | | | | |  |  |
| ^c^ Passage number used in experiment  ^d^ Has known family history of ALS | | | |  |  |  |

**Supplementary Table 2 – Quantitative ratios and corresponding changes**

| **Ratio** | **Region** | **Comparison** | **Mean difference** | **95.00% CI of diff.** | **Adj. p value** |
| --- | --- | --- | --- | --- | --- |
| Carbonyl ester / Total lipids | 1750-1725 cm^-1^ / 3000-2800 cm^-1^ | CTRL vs. MS | 0.021 | 0.0015 to 0.0395 | 0.0321 |
|  |  | CTRL vs. ALS | 0.014 | -0.005018 to 0.0330 | 0.1809 |
|  |  | MS vs. ALS | -0.007 | -0.0255 to 0.0124 | 0.6714 |
| Carbonyl ester / Acyl chain | 1750-1725 cm^-1^ / 1470-1430 cm^-1^ | CTRL vs. MS | 0.06 | 0.0207 to 0.0997 | 0.0022 |
|  |  | CTRL vs. ALS | 0.039 | -0.0008 to 0.0781 | 0.0561 |
|  |  | MS vs. ALS | -0.022 | -0.0611 to 0.0178 | 0.3763 |
| Carbonyl ester / Asymmetric CH3 | 1750-1725 cm^-1^ / 2972-2950 cm^-1^ | CTRL vs. MS | 0.021 | 0.0015 to 0.0395 | 0.0321 |
|  |  | CTRL vs. ALS | 0.014 | -0.0050 to 0.0329 | 0.1809 |
|  |  | MS vs. ALS | -0.007 | -0.0255 to 0.0124 | 0.6714 |
| Amide I /  Amide II | 1700-1580 cm^-1^ / 1580-1480 cm^-1^ | CTRL vs. MS | -0.089 | -0.1528 to -0.02517 | 0.005 |
|  |  | CTRL vs. ALS | -0.057 | -0.1205 to 0.0071 | 0.0893 |
|  |  | MS vs. ALS | 0.032 | -0.0314 to 0.0962 | 0.4311 |
|  |  |  |  |  |  |
|  |  |  |  |  |  |
|  |  |  |  |  |  |
| Amide I /  Amide A | 1700-1580 cm^-1^ / 3315-3260 cm^-1^ | CTRL vs. MS | -0.04 | -0.0839 to 0.0035 | 0.0765 |
|  |  | CTRL vs. ALS | 0.018 | -0.0253 to 0.0622 | 0.5545 |
|  |  | MS vs. ALS | 0.059 | 0.0149 to 0.1025 | 0.007 |
| Amide I /  Total lipids | 1700-1580 cm^-1^ / 3000-2800 cm^-1^ | CTRL vs. MS | -0.212 | -0.3549 to -0.0693 | 0.0028 |
|  |  | CTRL vs. ALS | -0.107 | -0.2500 to 0.0356 | 0.1694 |
|  |  | MS vs. ALS | 0.105 | -0.0378 to 0.2477 | 0.1816 |
| Amide I /  Carbonyl ester | 1700-1580 cm^-1^ / 1750-1725 cm^-1^ | CTRL vs. MS | -6.922 | -11.01 to -2.836 | 0.0007 |
|  |  | CTRL vs. ALS | -3.644 | -7.729 to 0.4422 | 0.0874 |
|  |  | MS vs. ALS | 3.278 | -0.8074 to 7.364 | 0.1341 |
| Carbonyl ester / Mixed region | 1750-1725 cm^-1^ / 1300-1000 cm^-1^ | CTRL vs. MS | 0.013 | 0.0058 to 0.0206 | 0.0004 |
|  |  | CTRL vs. ALS | 0.01 | 0.0025 to 0.0173 | 0.0067 |
|  |  | MS vs. ALS | -0.003 | -0.0106 to 0.0041 | 0.5263 |

Significant changes between groups were detected using one-way ANOVA post hoc Tukey test.
